# Development of Machine Learning Models with Blood‐based Digital Biomarkers for Diagnosis of Alzheimer's Disease

**DOI:** 10.1002/alz70856_099714

**Published:** 2025-12-25

**Authors:** Bin Jiao, Ziyu Ouyang, Shilin Luo, Beisha Tang, Zhigang Li, Lu Shen

**Affiliations:** ^1^ Xiangya Hospital, Central South University, Changsha, Hunan, China; ^2^ College of Information Science and Engineering, Northeastern University, Shenyang, Liaoning, China

## Abstract

**Background:**

Alzheimer's disease (AD) involves complex alterations in biological pathways, making comprehensive blood biomarkers crucial for accurate and earlier diagnosis. However, the cost‐effectiveness and operational complexity of method using blood‐based biomarkers significantly limit its availability in clinical practice.

**Methods:**

We developed low‐cost, convenient machine learning‐based with digital biomarkers (MLDB) using plasma spectra data to detect AD or mild cognitive impairment(MCI) from healthy controls (HCs) and discriminate AD from different types of neurodegenerative diseases. We included 1,324 individuals, including 293 with amyloid beta positive AD, 151 with mild cognitive impairment (MCI), 106 with Lewy body dementia (DLB), 106 with frontotemporal dementia (FTD), 135 with progressive supranuclear palsy (PSP) and 533 healthy controls (HCs).

**Result:**

Random forest classifier and feature selection procedures were used to select digital biomarkers. MLDB achieved area under the curves (AUCs) of 0.92 (AD vs HC, Sensitivity 88.2%, specificity 84.1%), 0.89 (MCI vs HC, Sensitivity 88.8%, specificity 86.4%), 0.83 (AD vs DLB, Sensitivity 77.2%, specificity 74.6%), 0.80 (AD vs FTD, sensitivity 74.2%, specificity 72.4%), and 0.93 (AD vs PSP, sensitivity 76.1%, specificity 75.7%). Digital biomarkers distinguishing AD from HC were negatively correlated with plasma *p*‐tau217 (r=‐0.22, *p* <0.05) and GFAP (r=‐0.09, *p* <0.05).

**Conclusion:**

The ATR‐FTIR (Attenuated Total Reflectance‐Fourier Transform Infrared) plasma spectra features can identify AD‐related pathological changes. These spectral features serve as digital biomarkers, significantly aiding in the early screening and diagnosis of AD.

